# A Formalized Design Process for Bacterial Consortia That Perform Logic Computing

**DOI:** 10.1371/journal.pone.0057482

**Published:** 2013-02-28

**Authors:** Weiyue Ji, Handuo Shi, Haoqian Zhang, Rui Sun, Jingyi Xi, Dingqiao Wen, Jingchen Feng, Yiwei Chen, Xiao Qin, Yanrong Ma, Wenhan Luo, Linna Deng, Hanchi Lin, Ruofan Yu, Qi Ouyang

**Affiliations:** 1 Peking University Team for the International Genetically Engineered Machine Competition (iGEM), Peking University, Beijing, China; 2 Center for Quantitative Biology and Peking-Tsinghua Joint Center for Life Sciences, Beijing, China; 3 The State Key Laboratory for Artificial Microstructures and Mesoscopic Physics, School of Physics, Peking University, Beijing, China; University of Akron, United States of America

## Abstract

The concept of microbial consortia is of great attractiveness in synthetic biology. Despite of all its benefits, however, there are still problems remaining for large-scaled multicellular gene circuits, for example, how to reliably design and distribute the circuits in microbial consortia with limited number of well-behaved genetic modules and wiring quorum-sensing molecules. To manage such problem, here we propose a formalized design process: (i) determine the basic logic units (AND, OR and NOT gates) based on mathematical and biological considerations; (ii) establish rules to search and distribute simplest logic design; (iii) assemble assigned basic logic units in each logic operating cell; and (iv) fine-tune the circuiting interface between logic operators. We *in silico* analyzed gene circuits with inputs ranging from two to four, comparing our method with the pre-existing ones. Results showed that this formalized design process is more feasible concerning numbers of cells required. Furthermore, as a proof of principle, an *Escherichia coli* consortium that performs XOR function, a typical complex computing operation, was designed. The construction and characterization of logic operators is independent of “wiring” and provides predictive information for fine-tuning. This formalized design process provides guidance for the design of microbial consortia that perform distributed biological computation.

## Introduction

Microbial consortia refer to a group of multiple interacting microbial populations to perform certain functions. They are ubiquitous in nature [1]–[9]. For instance, the human microbiome in gut assists to assimilate various substrates, harvest energy, and synthesize vitamins [1]; bacterial consortia naturally exist in soil are capable of degrading different hydrocarbon contaminations [2]. Learnt from natural microbial consortia, synthetic biologists started to create biological “machines” utilizing microbial consortia, aiming at designing and constructing novel functions. The recent examples include simultaneous fermentation of sugar mixtures including xylose and glucose [3], sensing and eradicating human pathogens [4], and designing different logic gates [5], [6]. Compared with using a single strain of bacterium to perform a designed function, engineering microbial consortia may lower metabolic burden for cells [10], [11], reduce crosstalk between cellular elements, and strengthen robustness to environmental fluctuations [12]. Moreover, cellular compartmentalization guarantees the reusability and modularity of genetic parts in the design of gene circuitry [5], [6], facilitating the scaling-up of synthetic gene circuits to accomplish more complicated tasks [13].

Synthetic gene circuits are often designed to perform Boolean logic function, either for application [14], [15], or as proof of concept [16]–[21]. Very recently, two attempts to exploit microbial consortia for distributive logic computation have been reported [5], [6]. They are dramatically different in design principle. Tamsir *et al.* engineered cells to carry a modular NOR gate with interchangeable input promoters and output signals, each acting as a logic-computing operator, wired by orthogonal quorum sensing molecules [5]. This design principle is quite electronic-like: any computing operation can be implemented by assembling standard NOR gates layer by layer. Regot *et al.*, however, constructed 16 different logic-computing operators, each encoded within a single yeast cell, dealing with one/two inputs either from environment or from another “operator”, and generating an output. This allows circuit design to be more feasible; it enables a circuit that requires fewer computing layers (thus fewer wiring molecules) [6].

In fact, there is a trade-off between standardization of logic modules (highlighted by Tamsir *et al.*) and wiring efficiency (highlighted by Regot *et al.*). Standardized logic operators allow synthetic biologists to predict system performance confidently, without taking into account too many other factors. However, more computing layers and wiring molecules are required. As a result, information processing will be slowed down and will inevitably exhaust limited “chemical wires” (e.g., quorum sensing molecules) [13]. On the other hand, although wiring efficiency could be guaranteed by using diverse logic modules, a lot more efforts must be spent on the construction, fine-tuning and characterization of individual logic modules.

**Figure 1:**
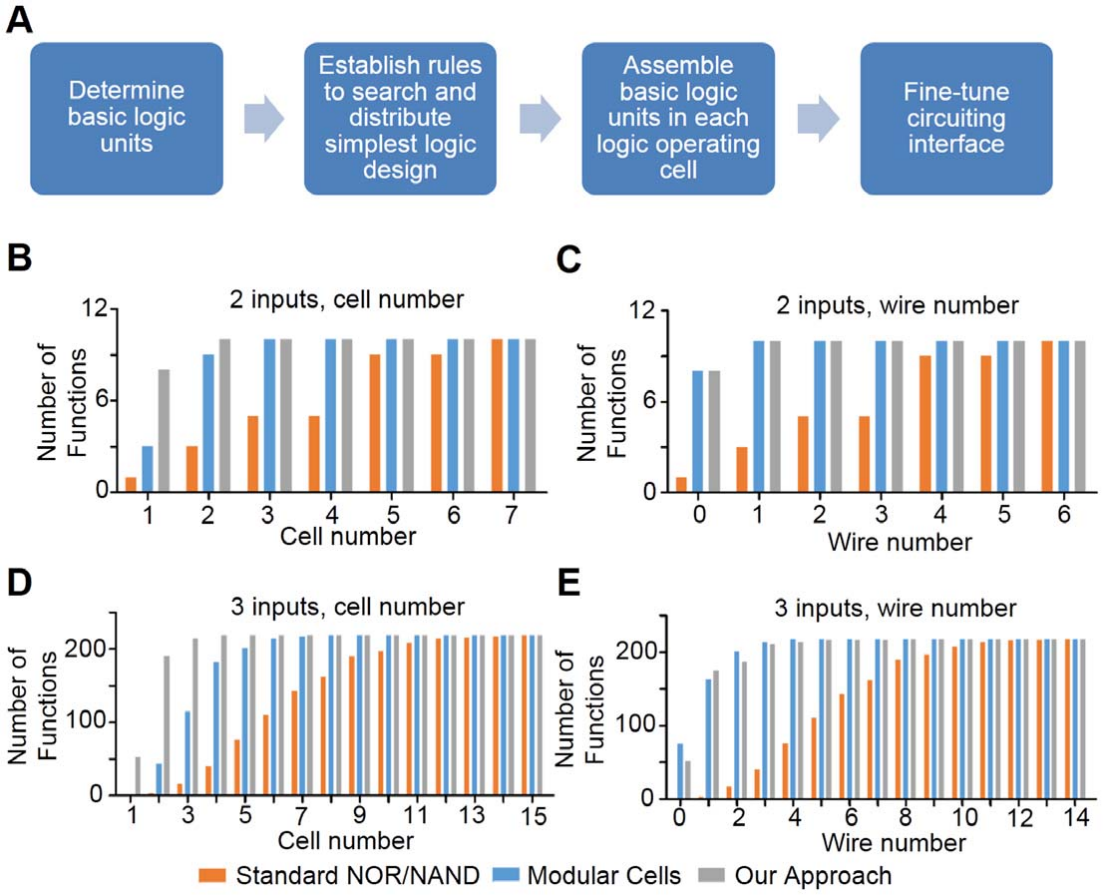
Work flow for the formalized design process and *in silico* analysis of different approaches in multicellular logic circuits. (A). Schematic view of the work flow for formalized design process. (B). Number of permissible 2-input 1-output Boolean functions versus the number of cells required for their implementation. Each bar represents number of functions that can be implemented within a certain number of cells. Different colors denote different approaches: orange for Standard NOR/NAND, blue for Modular Cells, and gray for our approach of combinational design. (C). Number of permissible 2-input 1-output Boolean functions versus the number of chemical wires required for their implementation. (D) and (E) show the results for 3-input 1-output Boolean functions.

In this work, we explore the possibility of a formalized design process to balance the trade-off between standardization of logic operators and wiring efficiency in engineered microbial consortia. In this process [Fig. 1(A)], AND, OR and NOT gates are chosen as the basic logic units and are combined to express desired computing operation as simplest logic. The simplest logic is distributed into separated logic operating cells according to carefully established rules. Logic operators (logic operating cells) were constructed and tested independently, and then combined to create a complete logic circuit though fine-tuning circuiting interface. As proof of principle, an *Escherichia coli* consortium that performs XOR gate operation, the operation usually considered as difficult to implement in synthetic biology, was designed and implemented.

## Results

### Formalization and Decomposition of Simplest Logic

We exploit computer-aided design to formalize the simplest logic for desired computing operation (see Text S1). The first step is to determine the basic logic units that have been proven to behave robustly in cells. For this consideration, modular genetic AND, OR and NOT gates (see Table S1) are chosen as basic logic operating units [17], [21]. They guarantee the design feasibility while avoiding laborious efforts on individual logic modules. These chosen logic gates form a functionally complete set; all possible truth tables can be expressed by combining them together [22].

Next we establish rules that guide the construction process where the three basic logic units are purposely selected and assembled in order to perform different logic computing. These rules are based on the following consideration. First, we speculate that the simplest design is usually the best in performance [23]. Therefore we established the first rule: (i) A combinational design should use fewest units (AND, OR or NOT gate). Sometimes, however, there might be more than one design with the same number of gates. We then took biological preference into consideration. Noticed that NOT gate and OR gate are easier to construct and cost less in cells, we set the second rule: (ii) Among the simplest designs, the one with more NOT and OR gates but fewer AND gates should be selected.

After the combinational design is determined, there remains another issue in making a good bacterial consortium, namely how to distribute the logic units into separated logic operating cells. We formalize two rules in this step of design. First, considering neighboring logic units in a cell can interact through tandem connections, and too many tandem layers would inevitably cause the system output to be sensitive to intrinsic noise and gene expression leakage, we prefer to compartmentalize those tandem layers into different cells. In this way, intrinsic noise could be suppressed by an average on wiring quorum-sensing molecules. This introduces our third rule: (iii) In every logic operator (i.e., logic-operating cell), tandem layers should be limited to no more than three; more necessary tandem layer should be distributed into different cells. This is because previous study revealed that in *E.coli* transcription regulatory network, the layer number of most sub-networks is no larger than three [24]. Finally, consider that parallel logic units could utilize a same input molecule. As regulatory proteins within a cell are limited (usually tens to hundreds of copies), promoters of parallel logic units will compete for the regulatory proteins [25]. As an undesirable result, performances of both paralleled units would be deteriorated. Therefore, at the aim of effectiveness in computation, we established another rule: (iv) Parallel unit should be reduced to minimum, or totally avoided if possible.

Combining together the four rules above, we *in silico* analyzed Boolean logic functions with two to four inputs and one output, and compared our method with two pre-existing methods, named as Standard NOR/NAND [5] or Modular Cells [6] in Figure 1, respectively. In the analysis, we focused on two characteristics: the number of permissible functions within given number of computing operators (namely number of cells) or that within given number of intercellular chemical wires. In the statistics, we excluded those functions that do not depend on all the inputs (e.g. constant functions or functions that only depend on the value of one input). Results showed that our approach has effectively reduced the number of computing operators required [Fig. 1(B, D), Figure S1]. For instance, using our approach, the number of permissible 3-input Boolean functions within two cells is 191 out of all 218, while for Standard NOR/NAND and Modular Cells, only 3 and 43 functions can be fulfilled within two cells, respectively. Also, four cells would be enough to implement all 3-input functions in our approach, while 15 cells and 8 cells are required for Standard NOR/NAND and Modular Cells approach, respectively. As for chemical wires, our approach is comparable with Modular Cells approach [Fig. 1(C, E)]: using our approach, 52 functions can be implemented without chemical wires, and 123 functions can be fulfilled with one chemical wire; and for Modular Cells approach the corresponding numbers are 75 and 88. Standard NOR/NAND apparently requires more wires compared with the others [Fig. 1(C, E)].

**Figure 2:**
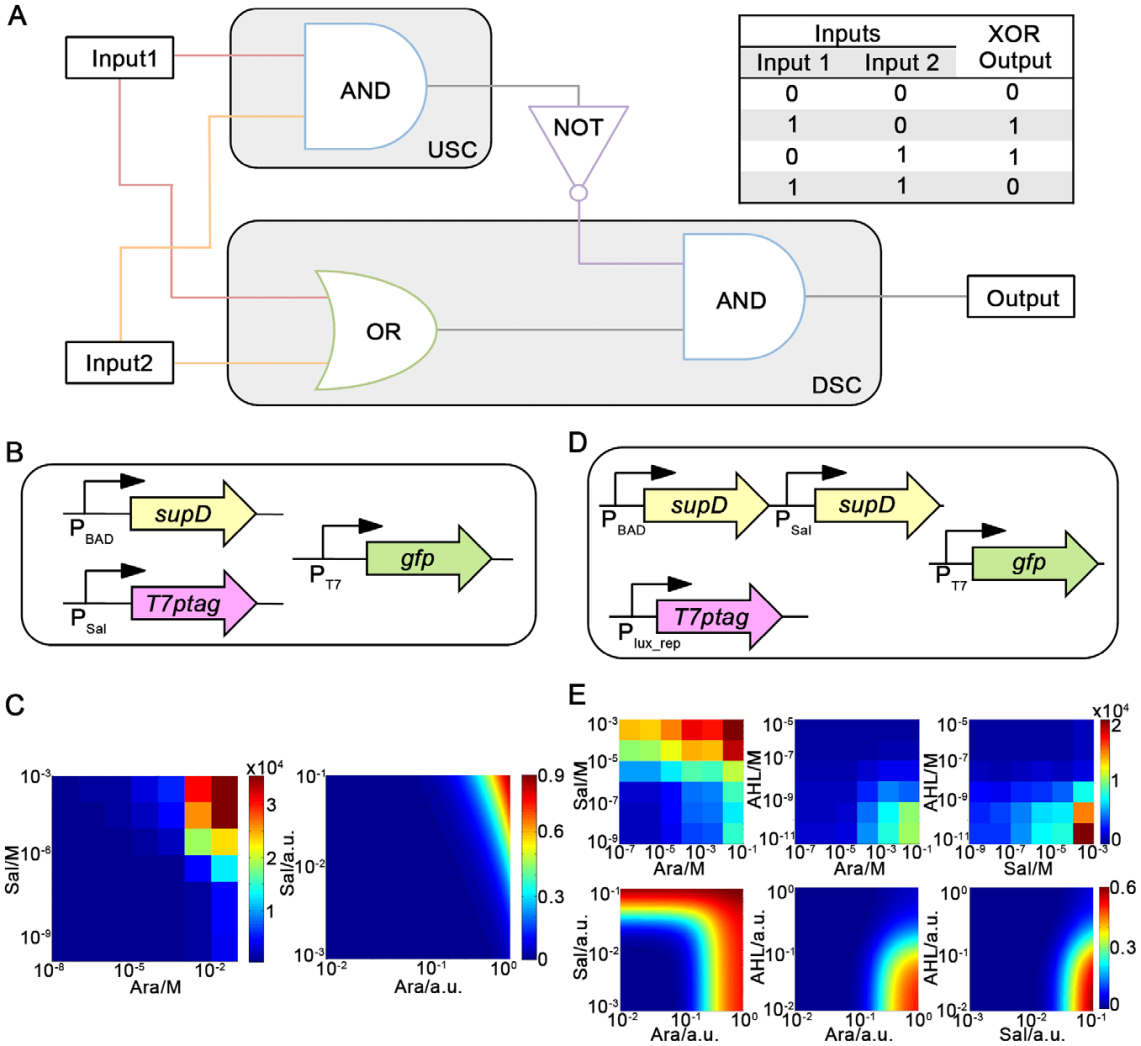
The simplest logic of XOR function, its distribution into separate logic operating cells, and characterization of two logic operators. (A). XOR function and its distribution. Left: The simplest logic of XOR gate expressed as the combination of basic logic units, according to the four rules in main text. XOR gate is distributed into two different logic-operating cells, USC and DSC. USC bears a genetic AND gate, with the output signal linked to DSC. DSC processes three inputs; two environmental inputs and an intermediate signal from USC. NOT gate does not belong to either cell, but is realized by transcription-inhibitory “chemical wire”. Such construction satisfies truth table of XOR gate presented in the right panel. (B). Gene circuit to characterize transfer function of USC. Only when both inputs exist, functional T7 polymerase would activate T7 promoter and produce output, GFP. (C). Left: Experimental results for transfer function of USC. Florescence was measured and normalized by cell density. The measured sets are for 10^−1^, 10^−2^, 10^−3^, 10^−4^, 10^−5^, 10^−6^, 10^−7^ and 10^−8^ M arabinose, and 10^−3^, 10^−4^, 10^−5^, 10^−6^, 10^−7^, 10^−8^, 10^−9^ and 10^−10^ M salicylate. Right: Corresponding simulation prediction. (D). Gene circuit of DSC. Both environmental inputs can drive the expression *supD* tRNA through corresponding promoters, composing an OR gate. With no AHL, *T7ptag* would be expressed, and thereby GFP could be produced when either arabinose or salicylate (or both of them) present. (E). Transfer function of DSC, showing combinations of every two inputs. Columns from left to right: arabinose and salicylate, arabinose and AHL, and salicylate and AHL. Upper panels show experimental data compared with corresponding simulation prediction (lower panel). The data are for 10^−1^, 10^−2^, 10^−3^, 10^−4^, 10^−5^, 10^−6^, and 10^−7^ M arabinose, 10^−3^, 10^−4^, 10^−5^, 10^−6^, 10^−7^, 10^−8^, and 10^−9^ M salicylate, and 10^−5^, 10^−6^, 10^−7^, 10^−8^, 10^−9^, 10^−10^, and 10^−11^ M AHL. AHL was artificially supplied to DSC rather than a signal from USC.

In formulation of two-input one-output functions, we found that they could all be implemented in a single cell without violation of above rules, except for XOR gate and EQUALS gate, where two logic operators (i.e., two logic-operating cells) are needed (see Figure S2). XOR and EQUALS functions have been rarely implemented within a single cell in previous attempts [5]. Therefore, as a proof of concept, we set out to biologically implement XOR function in an *E. coli* consortium consisting of two types of logic operating cells (the design of EQUALS gate is very similar to that of XOR gate, because their truth tables are mutually complementary). The circuit design of two logic operators is presented in Fig. 2(A), where two collaborating cells are named as Upstream Cell (USC) and Downstream Cell (DSC), respectively.

### Construction and Characterization of Logic Operators

Figure S3 shows schematic gene construction of three logic units (AND, OR and NOT gates). The genetic AND gate we used here based on the work of Anderson *el al.* [17]: an amber mutated T7 polymerase (*T7ptag*) can be rescued by *supD* tRNA, and as an output, genes of interest under T7 promoter would be transcribed only when functional T7 polymerase presents, which means *T7ptag* and *supD* (driven by two distinct input signals) must both exist (Figure S3). We have refined this AND gate and improved its performance [26]. OR gate is straightforward to construct: two promoters with the same downstream gene can serve as an OR gate. In prokaryotic genomes, such organization of promoters is very common. Previous study also utilized two tandem promoters with the same orientation as OR gate, but interference between tandem promoters may happen [5]. As for NOT gate, a repressor whose expression is under the control of input and turns off downstream promoter(s) is a conventional option [27]. In this contest, we also learnt from a previous work to convert a quorum-sensing transcriptional activator, such as LuxR, to a repressor [28]. Positioning *lux* box, the DNA binding site of LuxR, between (and partially overlapping) consensus -35 and -10 hexamers of promoter could readily implement such repressor (Figure S4). In the presence of AHL, LuxR would bind to the engineered promoter and further inhibit transcription initiation therein, while transcription moves on normally in absence of AHL. Similar design can be applied to other quorum-sensing regulators, such as RhlR (See Figure S4 and S5 for more information).

USC and DSC were constructed by assembling those three logic units in cells [Fig. 2(B) and Fig. 2(D)]. The transfer function of each logic-operating cell was characterized independently before circuiting them together. USC has almost the same construction as the original AND gate [26]; the only difference is that its output is LuxI [synthase of acyl homoserine lactone (AHL), a quorum-sensing molecule connecting USC and DSC]. For characterization, we substituted LuxI with GFP [see Fig. 2(B)], so that performance of AND gate could be quantified by measuring florescence. Fig. 2(C) depicts the performance of USC, including experimental data and simulation results (Details of simulation in Text S1, simulation parameters are shown in Table S2). USC works well as an AND gate, with signal-background ratio (the ratio of normalized florescence between “ON” states and “OFF” states) exceeding 100. Besides, we also measured the level of AHL when USC output was LuxI, and found the results consistent with those using GFP (Figure S6). Simulation for USC was consistent with experiment, and we obtained relevant parameters by fitting experimental data into simulation results. Those parameters are useful when we predict circuiting behavior later.

DSC has one more input, namely AHL, compared to USC. It is composed of an OR gate tandem connected with a downstream AND gate [Fig. 2(D)]. Due to cell compartmentalization, we are able to reuse the design of AND gate (*T7ptag* rescued by *supD* tRNA) as in USC, only by swapping input promoters and output signals according to system requirements. If necessary, such an AND gate module can still be adopted in other cells in more complex microbial consortia. For the transfer function of DSC, note that AHL has a repressing effect, which means the engineered promoter (called P_lux_rep_ later) would be at “ON” state when there is no existence of AHL. As a result, only when no AHL is in system and at least one of the other two signals (arabinose or salicylate) presents, output of DSC would be “ON”. In our initial experiment, strength of P_BAD_ seemed too low compared with P_Sal_ to generate significant output contrast. Therefore, we utilized a stronger P_BAD_ promoter by modifying its Aral1 site [29] (See Figure S7 for further information). After such improvement, combinations of arbitrary two inputs were tested, and all three experiments turned out satisfying. As shown in Fig. 2(E), when AHL is absent from the system, P_lux_rep_ is always on; therefore output of DSC is “ON” when either arabinose or salicylate (or both of them) exists. As a result, DSC behave as an OR gate [Fig. 2(E), left panel]. Under conditions either arabinose or salicylate is absent; however, DSC performs function of an NIMPLY gate: only with salicylate or arabinose but no AHL, output is “ON” [Fig. 2(E), middle and right panels]. Simulation was also performed for DSC; the corresponding parameters were determined through fitting.

### Fine-tuning of Circuiting Interface

**Figure 3:**
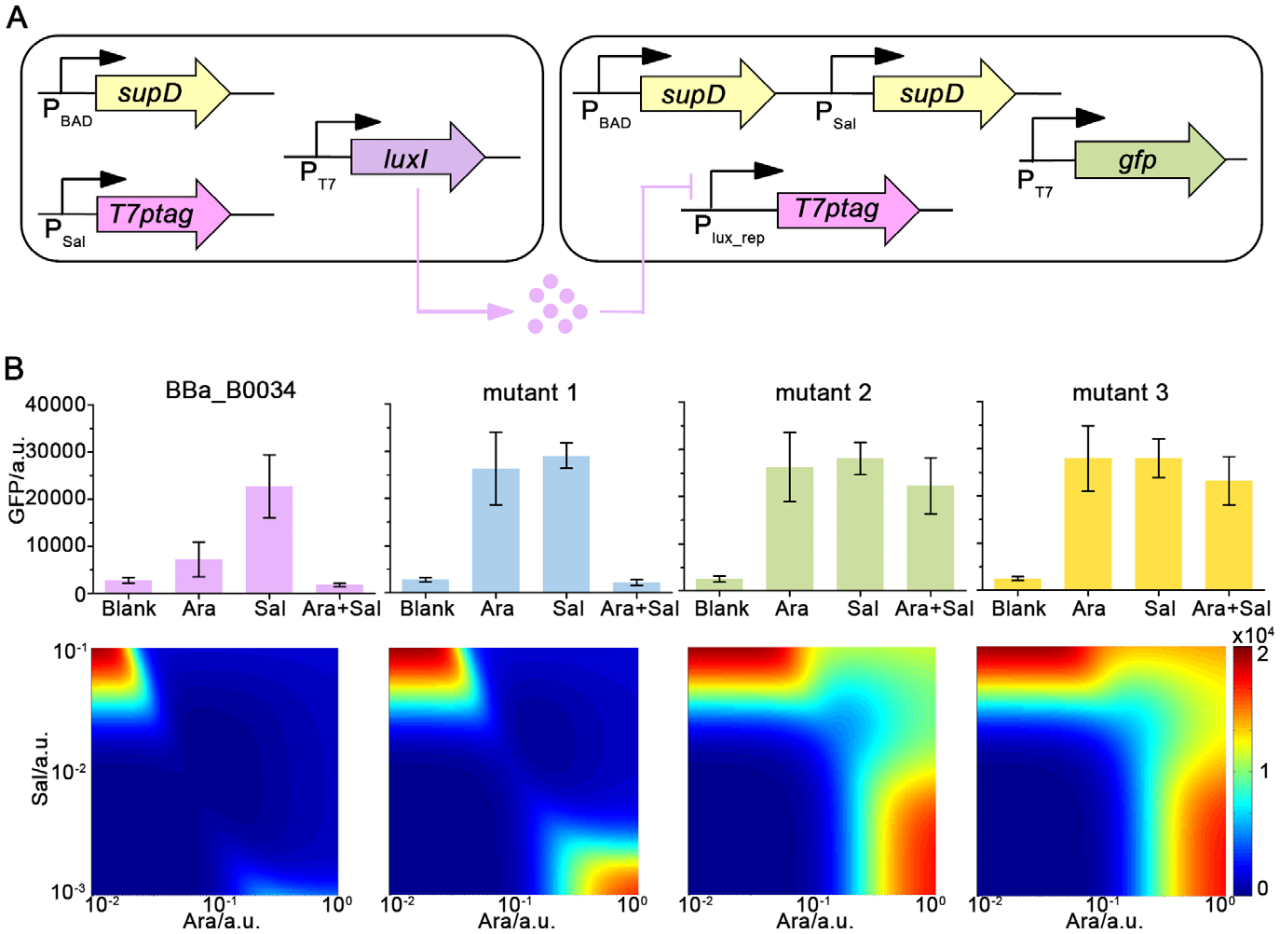
Fine-tuning of circuiting interface between USC and DSC. (A). Schematics of XOR-function gene circuit encoded within the entire microbial consortium. LuxI, a synthase of AHL, works as output of USC. AHL transduces a repressive signal to DSC. (B). Upper panels: experimental results using diluted filtrate from induced USC. Four histograms represent results for 4 different RBS sequences: AAAGAGGAGAAA (BBa_B0034), ATTAAAGTTGAGAAA (Mutant 1), GCTCCATCCCCG (Mutant 2), and GCTCCTCCGATC (Mutant 3), with RBS strength 9-, 108- and 150-fold attenuated, respectively, predicted by RBS Calculator. In each histogram, corresponding inputs are: (left to right) no inducers (blank), arabinose only (Ara), salicylate only (Sal), and both inducers (Ara+Sal). Error bars are calculated as mean ± s. d. Lower panels: phase diagrams of the entire circuit predicted by model using characterization data for individual logic operating cells.

After characterizing USC and DSC, we need to circuit them together according to our design [Fig. 2(A)]. Through quorum-sensing molecule AHL, USC and DSC can be coupled, where LuxI, LuxR, and promoter P_lux_rep_ together work as a transcription-inhibitory chemical wire for cell-cell signaling [Fig. 3(A)]. Fine-tuning process, however, was necessary. Hoping for rational fine-tuning, we utilized RBS Calculator as the tool. It could predict relative translation strength of a given RBS sequence and *in silico* design synthetic ribosome binding site (RBS) sequences with requested relative translation strength [30]. For this purpose, we use our initial construction [the RBS sequence was AAAGAGGAGAAA, numbered BBa_B0034 in Fig. 3(B)] as the reference. Filtrate from induced USC was used to culture DSC (filtrate was blended 1∶3 in volume with fresh Luria–Bertani broth, and arabinose and/or salicylate were also supplied as needed), and corresponding florescence of DSC were measured. Results showed that leakage expression of *luxI* in USC generated excessive AHL to repress P_lux_rep_ in DSC [Fig. 3(B), first panel in the upper row], indicating BBa_B0034 was too strong.

After getting the reference, we relied on our model simulation and RBS Calculator to get desired XOR function. With all other model parameters determined previously (see Text S1 for details in simulation, parameters are shown in Table S2), we fit experimental data of BBa_B0034 and searched for the optimal parameter. Our model predicted that XOR gate would perform best with approximate 10-fold attenuated translation strength of RBS prefixing *luxI* compared with BBa_B0034 (Figure S8). With the help of RBS calculator, we adopted three different RBS sequences with lower strengths predicted to be, respectively, 9-, 108- and 150-fold attenuated compared with BBa_B0034. Similar experimental measurements using filtrate from induced USC bearing each mutated RBS sequence were conducted to monitor the effect of RBS tuning (Table S3). Among 3 RBS sequences, Mutation 1 performed the best [Fig. 3(B), second panel], while the other two had too low strengths: with both arabinose and salicylate in the presence, neither of the latter two could enable USC to generate enough AHL to repress P_lux_rep_ in DSC. Additionally, model fitting using obtained experiment data revealed that RBS prefixing *luxI* had been actually attenuated by (8±3)-, (90±20)- and (110±20)-fold, respectively [Fig. 3(B)]. This result validates our rational fine-tuning process.

Taking all the above in account, we adopted Mutation 1 as the final design, with its sequence being ATTAAAGTTGAGAAA. The adapted USC still exhibited its property as AND gate, as shown in Figure S9. Due to the fact that the time scale of P_lux_rep_ response to AHL is shorter compared with that of cell growth (see Figure S5), time delay in signaling between two logic-operating cells would not be significant in experiments. Therefore, the best RBS determined in filtrate experiment is expected to work well when USC and DSC are cultured together.

### XOR Computation Operates Robustly

**Figure 4:**
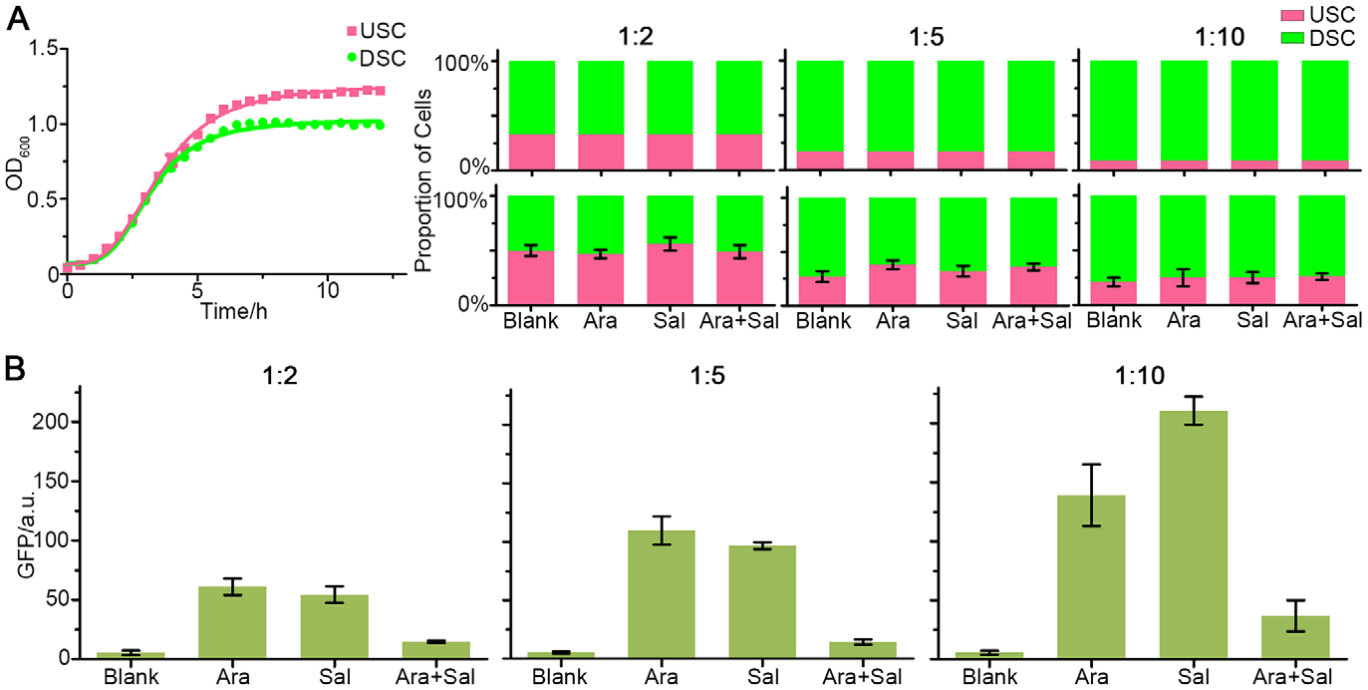
XOR computation operates robustly. (A). Growth curve of USC and DSC, showing OD600 as a function of time. Error bars are calculated as mean ± s. d. The lines are for guiding eyes. (B). Population proportions of USC and DSC under various conditions. Upper panel: initial population proportions at inoculation. Lower panel: corresponding population proportions after growth. Inducers were supplemented when inoculation. Cells were diluted and plated after growth, and colonies were counted to calculate population proportions. For all cases, *P*<0.001 (n = 3) for the differences in variations of USC population proportion under different treatments (Blank, Ara, Sal or Ara+Sal), using *χ^2^* test. (C). Microbial consortia with diverse initial proportions (1∶10, 1∶5 and 1∶2, respectively) all exhibited properties of XOR function. The results were measured by flow cytometry. Error bars are calculated as mean ± s. d.

USC and DSC were subsequently co-cultured as a microbial consortium to operate XOR computation. Notably, adjusting population proportions of inoculation between USC and DSC could affect, even disrupt XOR function because of unbalanced growth rates of logic operating cells. Our experimental results showed that the growth rate of USC was slightly faster compared with that of DSC [Fig. 4(A)], so population proportion would indeed vary during co-culture. Fortunately, as presented in Fig. 4(B), final population proportion was only determined by initial population proportion (regulated by inoculation), but almost unrelated to inducement conditions (blank, arabinose treatment, salicylate treatment, and both). To examine how population proportion correlates with XOR function, diverse population proportions between USC and DSC were applied in inoculation. Fig. 4(C) provides experimental data for three different inoculation ratios of USC:DSC, i.e., 1∶10, 1∶5 and 1∶2, whose final population proportions are close to 1∶4, 1∶3 and 1∶1 [Fig. 4(B)]. All three sets of inoculation ratios allow the microbial consortium to perform XOR function: when input is either only arabinose or only salicylate, output is high; but with both inputs or no input existing, output is low. Among all three sets, high contrast of XOR output is allowed (mostly higher than 10-fold difference). This result indicates that XOR computation is operated robustly despite varied population proportion in microbial consortium. Our simulation results also proved that population proportion is not a quite sensitive parameter in the system (See Figure S10).

## Discussion

To assemble more complex multicellular gene circuits, two general issues have to be addressed: how to design the input–output function of each logic operator, and how to couple the logic operators together [31]. To address the first issue, we established a formalized design process to reduce original function specification to individual logic operating cells harboring generic and readily available genetic parts/devices while avoiding the negative effect of parallel and tandem connection of genetic logic gates. For the second issue, we utilized transcription-inhibitory “chemical wires” engineered from bacterial quorum sensing system (Figure S4 and S5). Those engineered quorum-sensing repressors as NOT gates would be greatly useful when logic operating cells within a microbial consortium need to be coupled: a process of cell-cell signaling is at the same time a step of logic operation. This would bring about less metabolic burden and faster response due to the reduction of a procedure in transcription and translation. Following these considerations, we successfully constructed a microbial consortium that performs robust XOR computation. Such genetically encoded XOR gate circuit is robust in the sense that with population fluctuations, it could still function as expected.

Broadly speaking, since the construction of toggle switch and repressilator [32], [33], diverse genetic parts, devices, and circuits have been built using bottom-up approach [13]. Meanwhile top-down design is rapidly emerging [34]. Synthetic biology now comes to the stage of formalized design comprising both top-down decomposition and bottom-up assembly [13]. In this aspect, the formalized design process in this study essentially provides a good example.

## Methods

### Strains and Plasmids

All experiments were performed using *E. coli* strain DH5α. Plasmid construction was conducted via iGEM BioBrick standard assembly (http://openwetware.org/wiki/Synthetic_Biology:BioBricks/3A_assembly). All plasmids in our experiments are shown in Figure S11.

### Media, Chemicals, and Other Reagents

Strains were grown in LB liquid medium or on agar plates (1.5% agar), supplemented by antibiotics, ampicillin(50 µg/ml), kanamycin(10 µg/ml), and/or tetracycline(10 µg/ml). Inducers used were arabinose, salicylate and 3OC6-HSL. Reagents were purchased from Sigma-Aldrich. All restriction enzymes were from New England Biolabs, unless otherwise indicated. Primers were chemically synthesized at BGI Sequencing.

### RBS Strength Prediction

Strength prediction of RBS sequences prefixing *luxI* was conducted using online RBS calculator v1.1: Reverse Engineer RBSs (https://salis.psu.edu/software/reverse).

### Transfer Function Characterization

Cells harboring plasmids were separately incubated overnight (no less than 15 h) in 3 ml of LB broth medium (37°C, 250 r. p. m. shaking) in tubes without the presence of inducers. The cultures were then diluted 20-fold into 200 µl fresh LB broth medium with appropriate inducers in each well of a 96-well plate (2 ml in volume of each well) and incubated for additional 12 h before final pelleting and resuspension in 200 µl PBS (phosphate buffer saline) solution for micro-plate reader, or 10-fold dilution in PBS for cytometry analysis.

### Micro-plate Reader

All samples were measured florescence intensity using an exciting light of 470 nm and emitting light of 509 nm. Final florescence data were normalized by an OD600 value. Each sample contains 100 µl PBS solution in a well of micro-plate. All data were obtained using TECAN infinite M200.

### Flow Cytometry

All data contained at least 30,000 events. Mean value of the fluorescence distributions were calculated, and auto-fluorescence value of *E. coli* DH5α cells harboring no plasmid was subtracted before reporting the resulted fluorescence values. Data were obtained using BD LSR II Special Order System.

### Plating and Counting

After chemical inducement, cultures of mixed cells were diluted 100,000-fold and plated on agar plates applied with appropriate antibiotics and salicylate. Then the plates were incubated under 37°C for 15 h, and colonies on plates were counted. Surface of agar plate was divided into 9 grids, of which 5 were selected for counting (See Figure S12). USCs constitutively express RFP, and DSCs would express GFP due to salicylate on plates.

### Search of Simplest Logic Design

For the search of simplest logic, we used breadth-first search (BFS) method to exhaust possible combinations of certain logic units, thus generating the simplest one. Further, Greedy Algorithm was applied to divide the units into different cells.

### Numerics

All simulations were performed using MATLAB Version 7.13.0.564(R2011b) (Mathworks), and so were the experimental results in Fig. 2. Experimental results in Fig. 3 and 4 were processed using Graph Pad Prism 5, and figures in Supporting Information were processed using Graph Pad Prism 5 and Origin Pro 8. Logic gate assembly was through a cpp program on Qt platform, whose code is available in Text S1.

## Supporting Information

Figure S1 ***In silico*** **analysis of different approaches in multicellular logic circuits with 4 inputs and 1 output.** Due to the limitation of computation capacity, we cannot exhaust all 4-input functions. So we just calculated functions which can be implemented with no more than 7 cells and 4 chemical wires. Again, our approach outweighed others in the number of computing operators, and remained comparative chemical wires. (A). Number of permissible 4-input 1-output Boolean functions versus the number of cells required for their implementation. (B). Number of permissible 4-input 1-output Boolean functions versus the number of chemical wires required for their implementation.
(TIF)

Figure S2 **Simplest logic of all sixteen 2-input, 1-output logic gates expressed as combination of basic logic units (AND, OR and NOT gates).** The simplest logic was established using computer-aided design with our program according to the four rules in main text. All gate functions can be implemented in a single cell, except for XOR and EQUALS gates.
(TIF)

Figure S3 **Biological implementation of genetic AND, OR and NOT gates.** AND gate (A): an amber mutated T7 polymerase rescued by *supD* tRNA. OR gate (B): two promoters with the same downstream gene. NOT gate (C): engineered quorum-sensing repressor.
(TIF)

Figure S4 **Working mechanism of a quorum-sensing repressor.** To convert a quorum-sensing transcriptional activator, such as LuxR, to a repressor, we positioned *lux* box, the DNA binding site of LuxR, between (and partially overlapping) consensus -35 and -10 hexamers of promoter, so that the binding of LuxR to *lux* box would repress the accessibility of RNA polymerase to promoter. Similar designs can be applied to RhlR and other LuxR-family transcriptional activators. (A). Quorum-sensing transcriptional repressor, without AHL. (B). Quorum-sensing transcriptional repressor, with AHL in the presence.
(TIF)

Figure S5 **Dose response and time course of the modified repressive** ***lux*** **and** ***rhl*** **promoters.** Coding sequence of fast-degradation GFP (GFP translationally fused with LVA, a fast-degradation protein tag) was exploited as the reporter and then induced by gradient concentrations of 3OC6HSL or C4HSL, and afterwards normalized florescence was measured. (A). Dose response of P_lux_rep_ with different inducing time duration. The data sets are for 10^−5^, 10^−6^, 10^−7^, 10^−8^, 10^−9^, 10^−10^, 10^−11^, 10^−12^ and 10^−13^ M 3OC6HSL. Error bars are calculated as mean ± s. d. (B). Time course of P_lux_rep_, with 10^−5^ M 3OC6HSL inducing. Error bars are calculated as mean ± s. d. P_lux_rep_ has a quite fast response to 3OC6HSL, so that co-cultured USC and DSC were capable of transmitting signals within a short time. (C). Dose response of modified *rhl* repressive promoter. The data sets are for 10^−6^, 5×10^−7^, 3.5×10^−7^, 10^−7^, 5×10^−8^, 2×10^−8^, 10^−8^, 10^−9^, and 10^−10^ M C4HSL. Error bars are calculated as mean ± s. d. (D). Time course of modified *rhl* repressive promoter, with 10^−6^ M C4HSL inducing. Error bars are calculated as mean ± s. d. Lines in all subfigures are for guiding eyes.
(TIF)

Figure S6 **Transfer function of USC, when AHL levels are measured as the output.** USC was first induced with different concentrations of inducers, and filtrate from induced USC was used to culture cells bearing P_lux_rep_ with *gfp* downstream (filtrate was blended 1∶3 in volume with fresh Luria–Bertani broth). Afterwards, corresponding florescence was measured through a micro-plate reader. The florescence has a negative correlation with AHL concentration expressed by USC: the higher AHL concentration, the more P_lux_rep_ is repressed, and thus the less florescence in cells. (A). Experimental results. The data are for 10^−1^, 10^−2^, 10^−3^, 10^−4^, 10^−5^, 10^−6^, 10^−7^ and 10^−8^ M arabinose, and 10^−3^, 10^−4^, 10^−5^, 10^−6^, 10^−7^, 10^−8^, 10^−9^ and 10^−10^ M salicylate. (B). Corresponding simulation prediction.
(TIF)

Figure S7 **Transfer function of P_BAD_ (A), adapted stronger P_BAD_ version (B) and P_Sal_ (C).** Cells bearing each promoter with *gfp* downstream were induced, and florescence was measured through a micro-plate reader. The data are for 10^−1^, 10^−2^, 10^−3^, 10^−4^, 10^−5^, 10^−6^, 10^−7^, 10^−8^ and 10^−9^ M arabinose, and 10^−3^, 10^−4^, 10^−5^, 10^−6^, 10^−7^, 10^−8^, 10^−9^, 10^−10^ and 10^−11^ M salicylate. As shown in the figures (A) and (B), the adapted P_BAD_ is stronger, compared with the original. The lines are for guiding eyes.
(TIF)

Figure S8 **Model prediction for fine-tuning of circuiting interface.** By adjusting *luxI* translation strength, different phase diagrams have been obtained. (A). Phase diagram for the original RBS, of which the sequence is AAAGAGGAGAAA. The other nine subfigures show phase diagrams with reduced translation strength in simulation. From (B) to (J), translation strengths are attenuated by 2, 5, 10, 20, 50, 100, 200, 500, and 1000 folds, respectively. And their corresponding signal-background ratios (the ratios of protein expression level between “ON” state and “OFF” state) are approximate 6, 9, 10, 8, 3, 2, 1, 0.9 and 0.8 in simulation. So we predict with modeling results that approximate 10-fold attenuated translation strength of RBS prefixing *luxI* would improve the circuiting.
(TIF)

Figure S9 **Transfer function of adapted USC with the RBS prefixing** ***luxI*** **changed to ATTAAAGTTGAGAAA.** AHL levels are measured as the output. Experiment protocols are the same as described in the legend of Figure S6. The adapted USC still functions as an AND gate: only when both arabinose and salicylate exist, AHL would be expressed. (A). Experimental results. The data are for 10^−1^, 10^−2^, 10^−3^, 10^−4^, 10^−5^, 10^−6^, 10^−7^ and 10^−8^ M arabinose, and 10^−3^, 10^−4^, 10^−5^, 10^−6^, 10^−7^, 10^−8^, 10^−9^ and 10^−10^ M salicylate. (B). Corresponding simulation prediction.
(TIF)

Figure S10 **XOR gate operates robustly under population fluctuation in simulation.** In simulation, we changed the population proportion of USC and DSC, and found that within a quite large range (from USC:DSC = 1∶20 to USC:DSC = 1∶1), the system could always exhibit a high signal-background ratio. (A). USC:DSC = 1∶20, (B). USC:DSC = 1∶10, (C). USC:DSC = 1∶5, (D). USC:DSC = 1∶2, and (E). USC:DSC = 1∶1.
(TIF)

Figure S11 **Plasmid constructions of USC and DSC.** (A). Construction of USC for XOR function. The plasmid bearing RFP was used for flow cytometry assay. (B). Construction of DSC for XOR function. (C). Construction of USC used to measure its transfer functions. (D). Constructions used to measure AHL expression of USC.
(TIF)

Figure S12 **Plating and counting.** When counting population proportions (in Methods), surface of agar plate was divided into 9 grids, of which 5 were selected for counting. We did not count the four regions at the corner with relative small area.
(TIF)

Table S1 **Truth tables for AND, OR and NOT gates.**
(PDF)

Table S2 **Parameters and their corresponding values used in simulation.**
(PDF)

Table S3 **Primers designed for mutagenesis of Ribosome Binding Site sequence prefixing** ***luxI*** **.**
(PDF)

Text S1 **Model for logic circuits, supplementary materials and methods.**
(PDF)
