# Supplementary figures and images for: A Formalized Design Process for Bacterial Consortia That Perform Logic Computing
Source: PLoS One. 2013 Feb 28;8(2):e57482. doi: 10.1371/journal.pone.0057482 (PMC3585339; doi:10.1371/journal.pone.0057482)

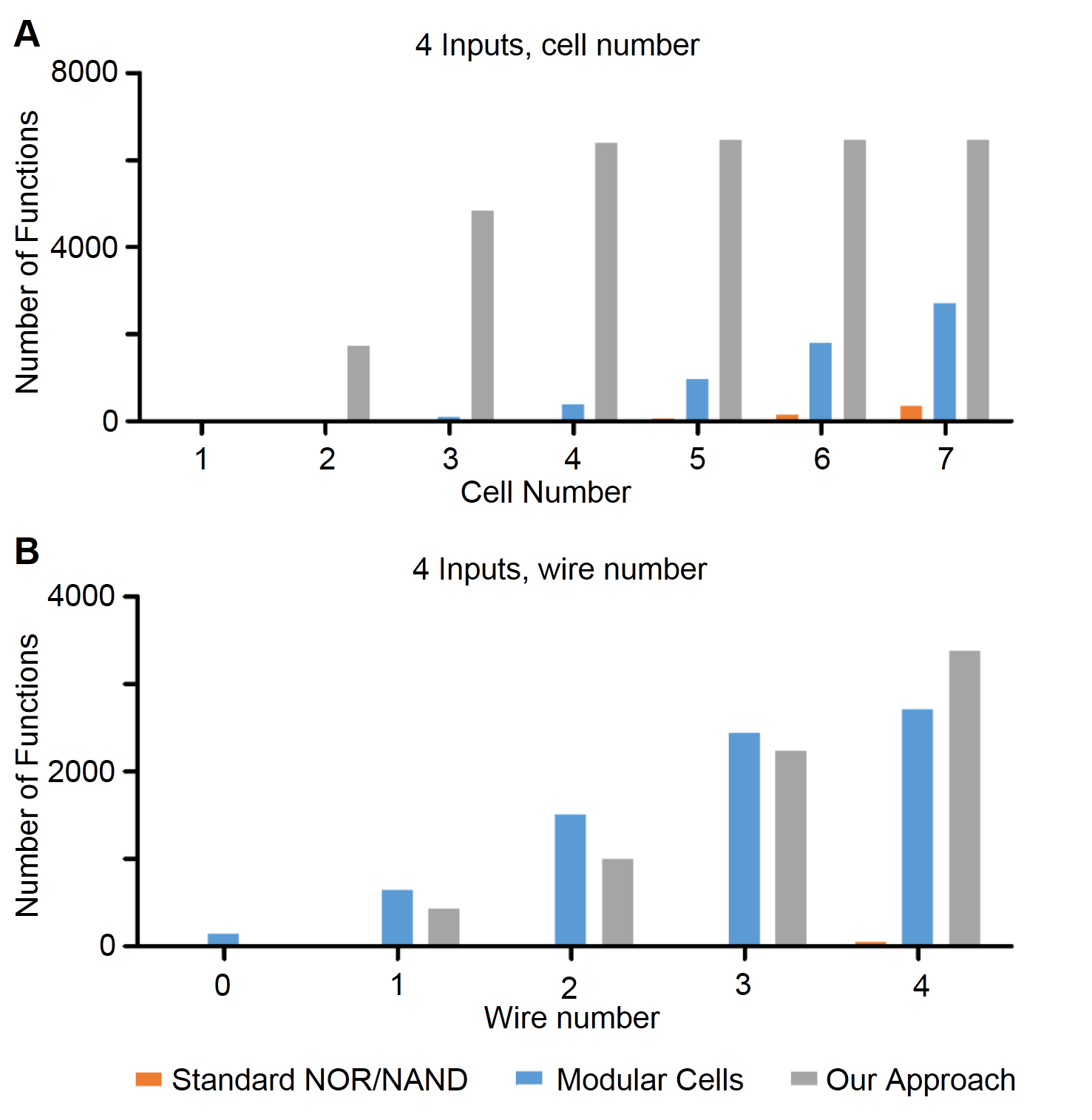

Supplement: Figure S1 — In silico analysis of different approaches in multicellular logic circuits with 4 inputs and 1 output. Due to the limitation of computation capacity, we cannot exhaust all 4-input functions. So we just calculated functions which can be implemented with no more than 7 cells and 4 chemical wires. Again, our approach outweighed others in the number of computing operators, and remained comparative chemical wires. (A). Number of permissible 4-input 1-output Boolean functions versus the number of cells required for their implementation. (B). Number of permissible 4-input 1-output Boolean functions versus the number of chemical wires required for their implementation. (TIF) [file pone.0057482.s001.tif]

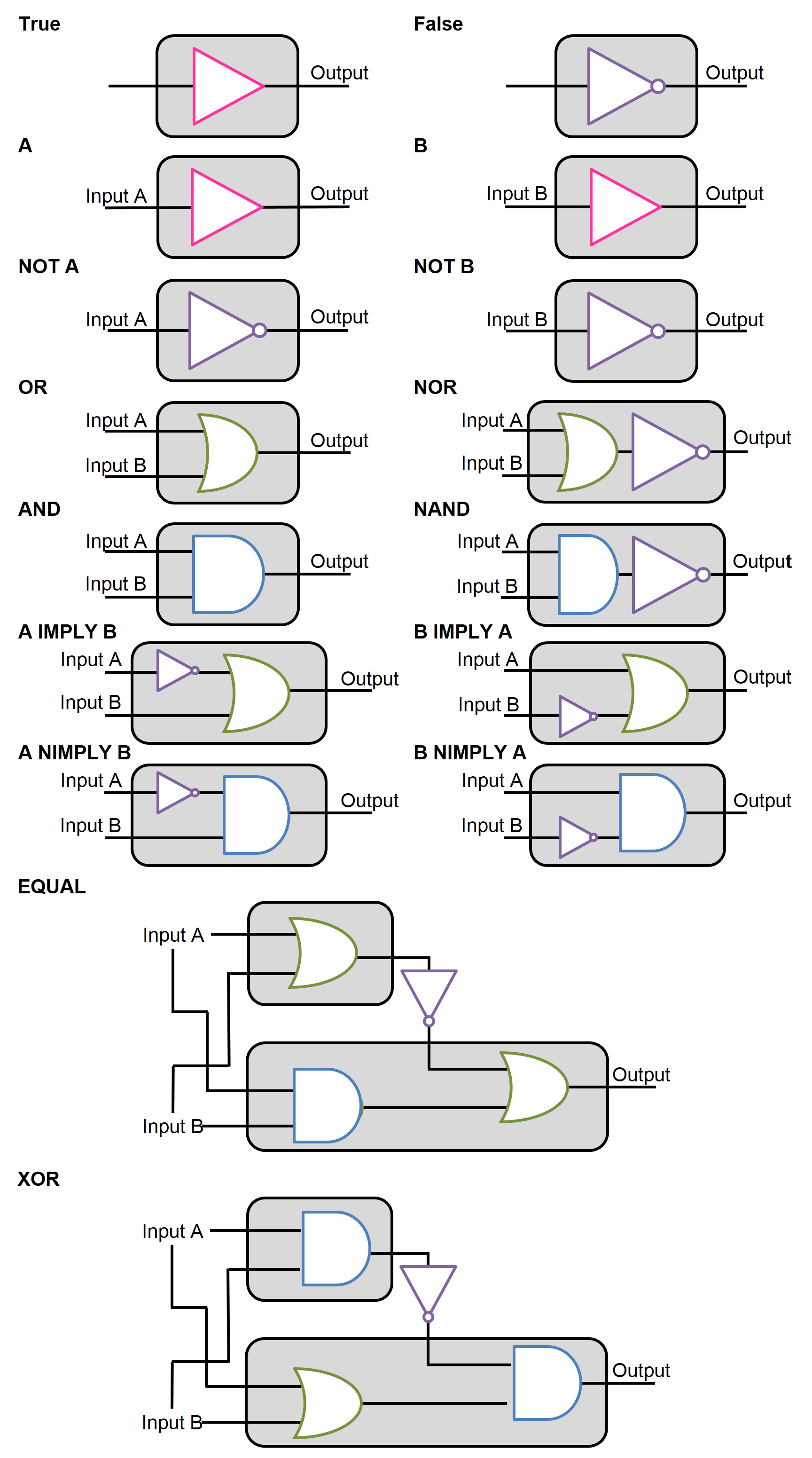

Supplement: Figure S2 — Simplest logic of all sixteen 2-input, 1-output logic gates expressed as combination of basic logic units (AND, OR and NOT gates). The simplest logic was established using computer-aided design with our program according to the four rules in main text. All gate functions can be implemented in a single cell, except for XOR and EQUALS gates. (TIF) [file pone.0057482.s002.tif]

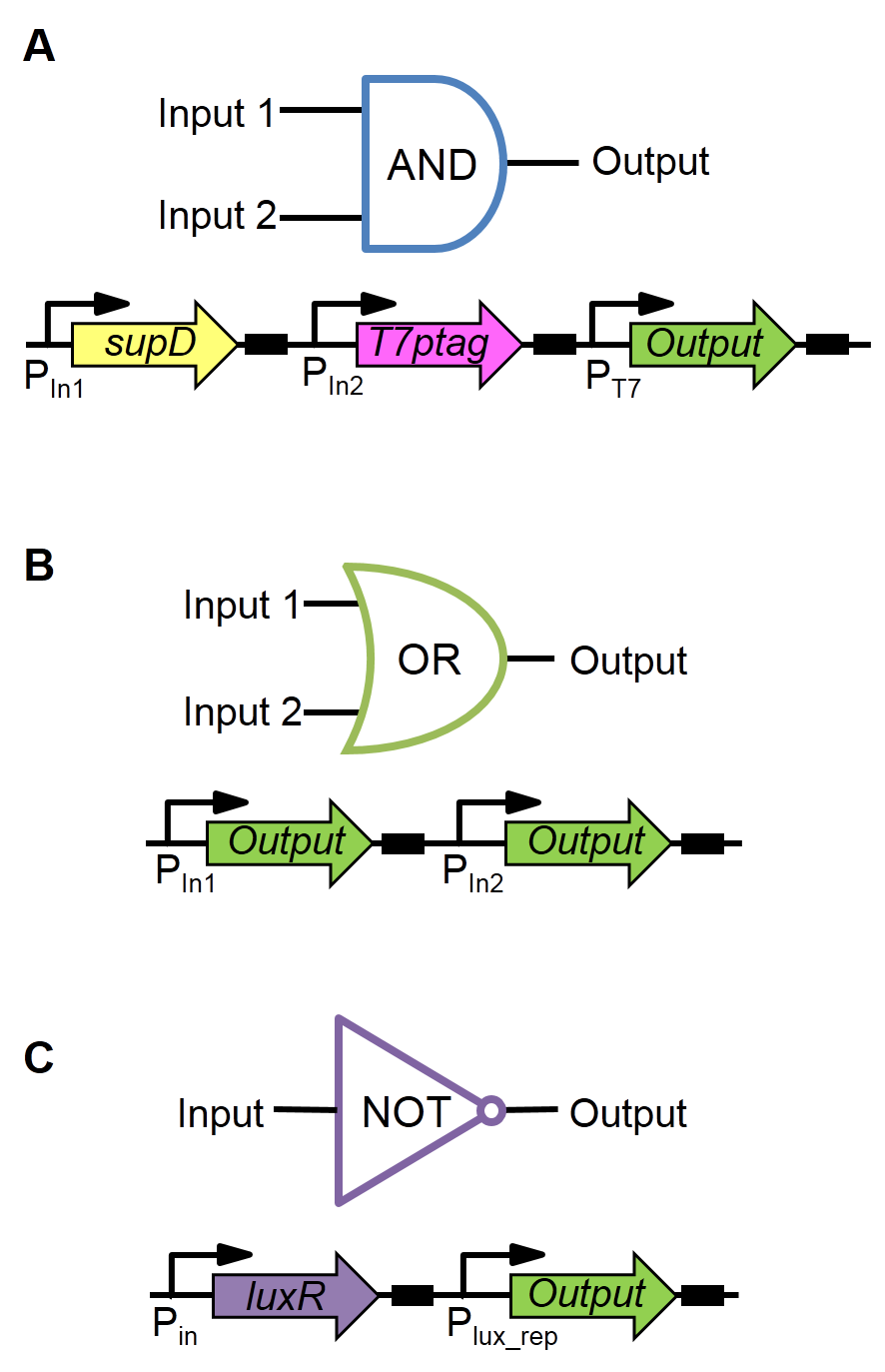

Supplement: Figure S3 — Biological implementation of genetic AND, OR and NOT gates. AND gate (A): an amber mutated T7 polymerase rescued by supD tRNA. OR gate (B): two promoters with the same downstream gene. NOT gate (C): engineered quorum-sensing repressor. (TIF) [file pone.0057482.s003.tif]

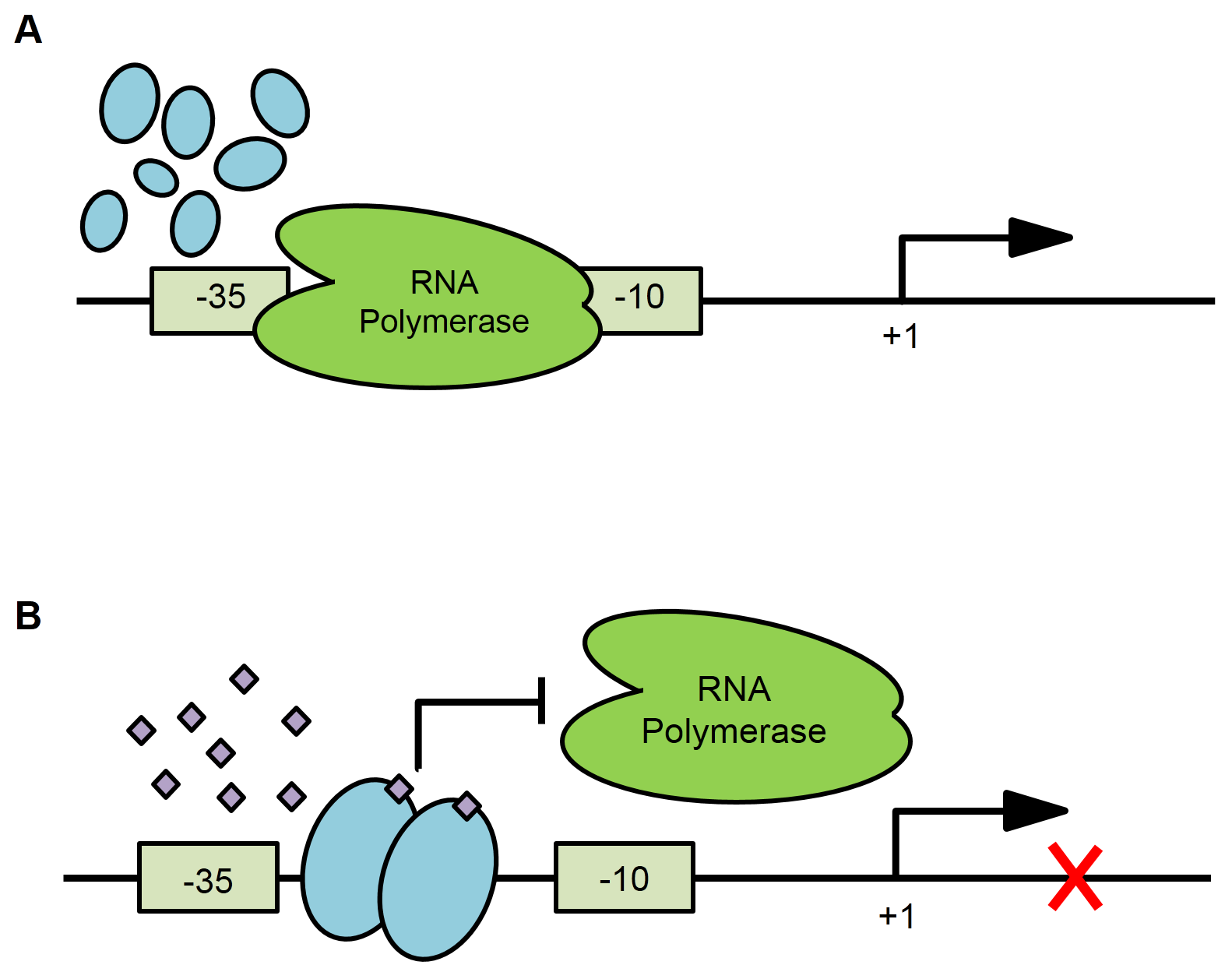

Supplement: Figure S4 — Working mechanism of a quorum-sensing repressor. To convert a quorum-sensing transcriptional activator, such as LuxR, to a repressor, we positioned lux box, the DNA binding site of LuxR, between (and partially overlapping) consensus -35 and -10 hexamers of promoter, so that the binding of LuxR to lux box would repress the accessibility of RNA polymerase to promoter. Similar designs can be applied to RhlR and other LuxR-family transcriptional activators. (A). Quorum-sensing transcriptional repressor, without AHL. (B). Quorum-sensing transcriptional repressor, with AHL in the presence. (TIF) [file pone.0057482.s004.tif]

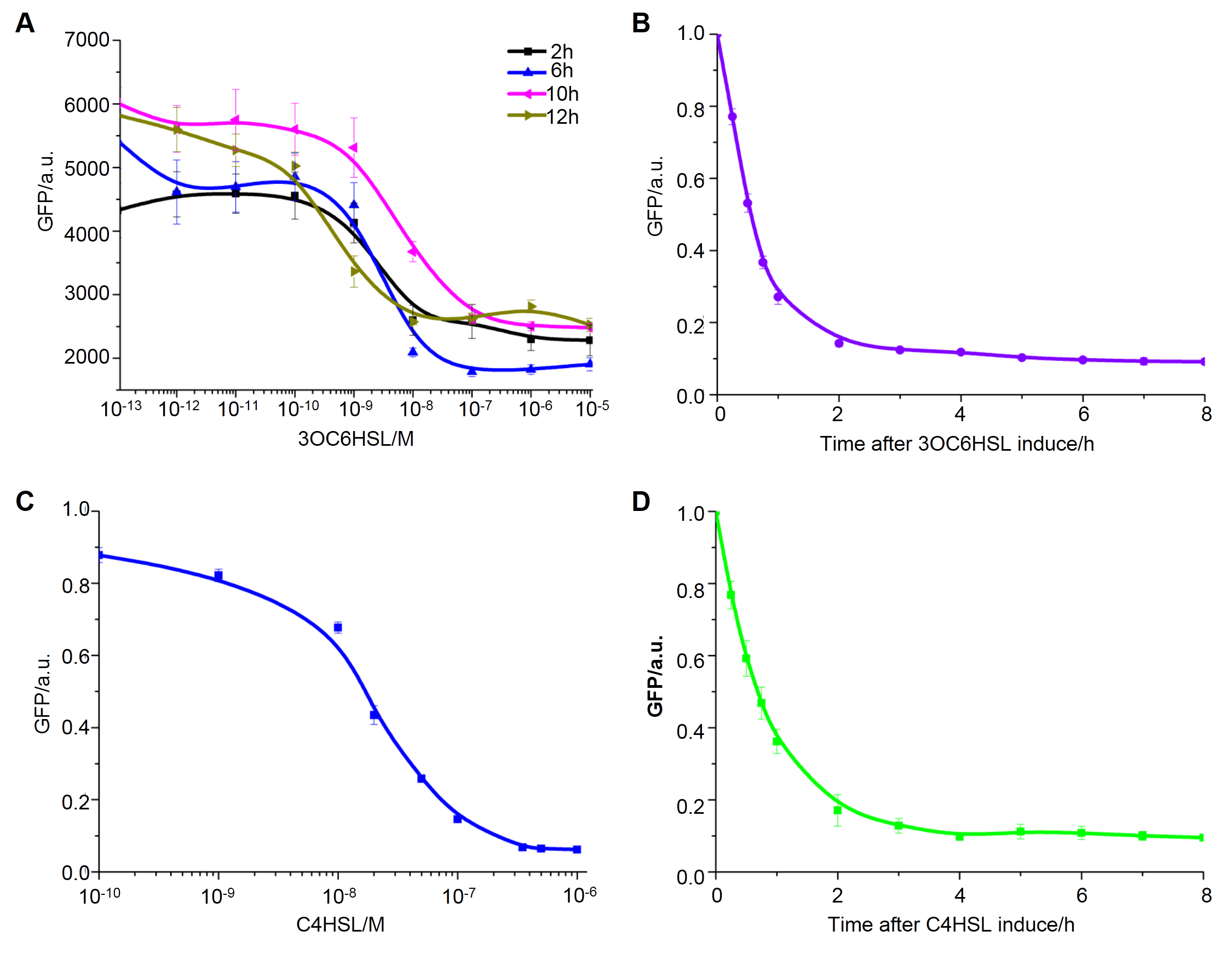

Supplement: Figure S5 — Dose response and time course of the modified repressive lux and rhl promoters. Coding sequence of fast-degradation GFP (GFP translationally fused with LVA, a fast-degradation protein tag) was exploited as the reporter and then induced by gradient concentrations of 3OC6HSL or C4HSL, and afterwards normalized florescence was measured. (A). Dose response of Plux_rep with different inducing time duration. The data sets are for 10−5, 10−6, 10−7, 10−8, 10−9, 10−10, 10−11, 10−12 and 10−13 M 3OC6HSL. Error bars are calculated as mean ± s. d. (B). Time course of Plux_rep, with 10−5 M 3OC6HSL inducing. Error bars are calculated as mean ± s. d. Plux_rep has a quite fast response to 3OC6HSL, so that co-cultured USC and DSC were capable of transmitting signals within a short time. (C). Dose response of modified rhl repressive promoter. The data sets are for 10−6, 5×10^−7^, 3.5×10^−7^, 10−7, 5×10^−8^, 2×10^−8^, 10−8, 10−9, and 10−10 M C4HSL. Error bars are calculated as mean ± s. d. (D). Time course of modified rhl repressive promoter, with 10−6 M C4HSL inducing. Error bars are calculated as mean ± s. d. Lines in all subfigures are for guiding eyes. (TIF) [file pone.0057482.s005.tif]

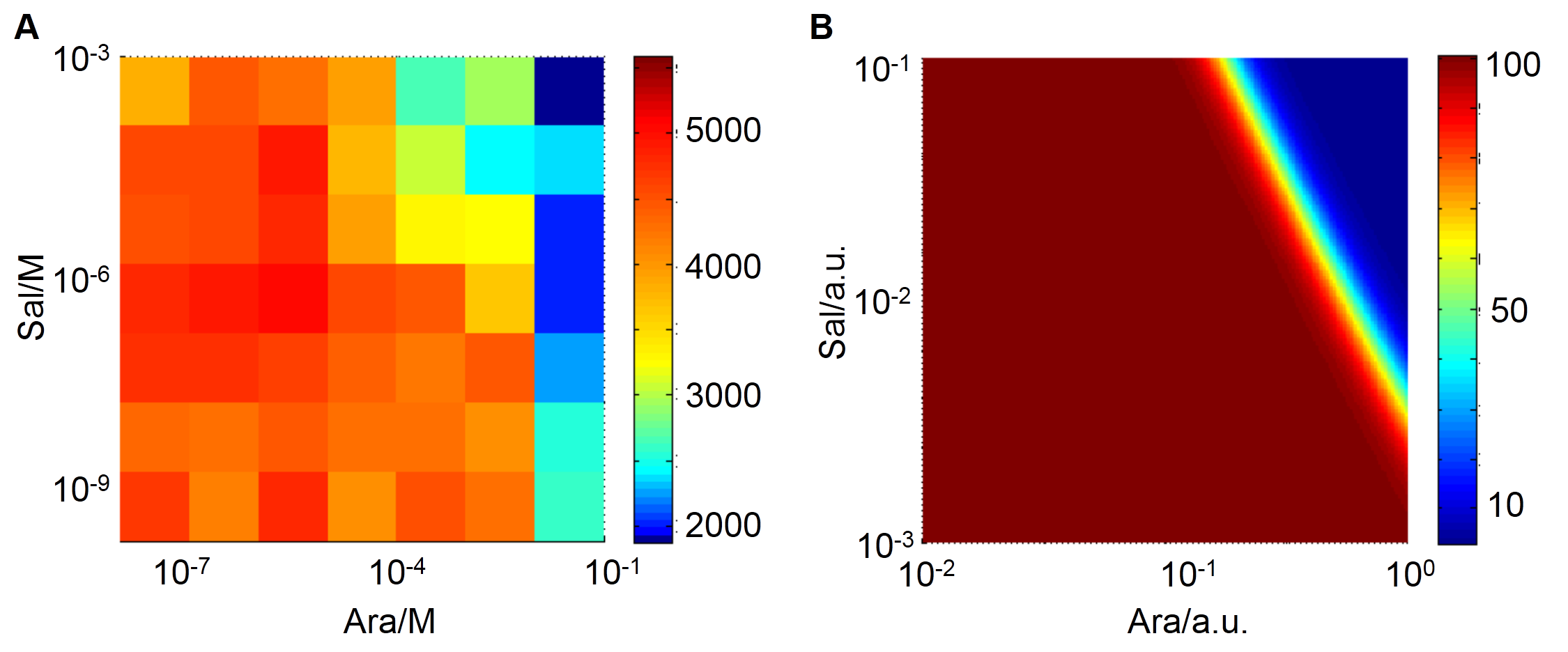

Supplement: Figure S6 — Transfer function of USC, when AHL levels are measured as the output. USC was first induced with different concentrations of inducers, and filtrate from induced USC was used to culture cells bearing Plux_rep with gfp downstream (filtrate was blended 1∶3 in volume with fresh Luria–Bertani broth). Afterwards, corresponding florescence was measured through a micro-plate reader. The florescence has a negative correlation with AHL concentration expressed by USC: the higher AHL concentration, the more Plux_rep is repressed, and thus the less florescence in cells. (A). Experimental results. The data are for 10−1, 10−2, 10−3, 10−4, 10−5, 10−6, 10−7 and 10−8 M arabinose, and 10−3, 10−4, 10−5, 10−6, 10−7, 10−8, 10−9 and 10−10 M salicylate. (B). Corresponding simulation prediction. (TIF) [file pone.0057482.s006.tif]

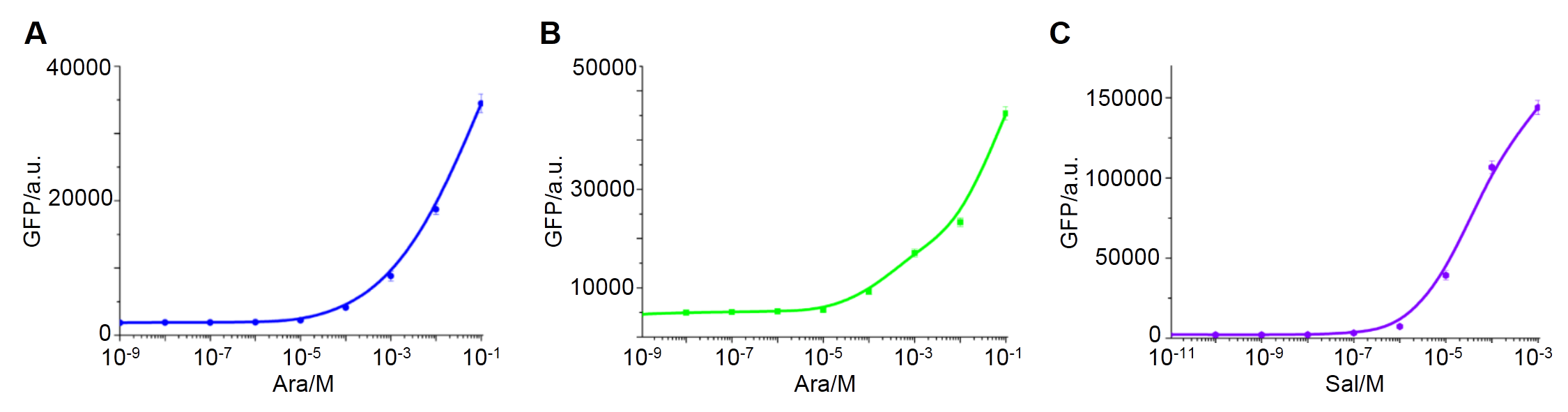

Supplement: Figure S7 — Transfer function of PBAD (A), adapted stronger PBAD version (B) and PSal (C). Cells bearing each promoter with gfp downstream were induced, and florescence was measured through a micro-plate reader. The data are for 10−1, 10−2, 10−3, 10−4, 10−5, 10−6, 10−7, 10−8 and 10−9 M arabinose, and 10−3, 10−4, 10−5, 10−6, 10−7, 10−8, 10−9, 10−10 and 10−11 M salicylate. As shown in the figures (A) and (B), the adapted PBAD is stronger, compared with the original. The lines are for guiding eyes. (TIF) [file pone.0057482.s007.tif]

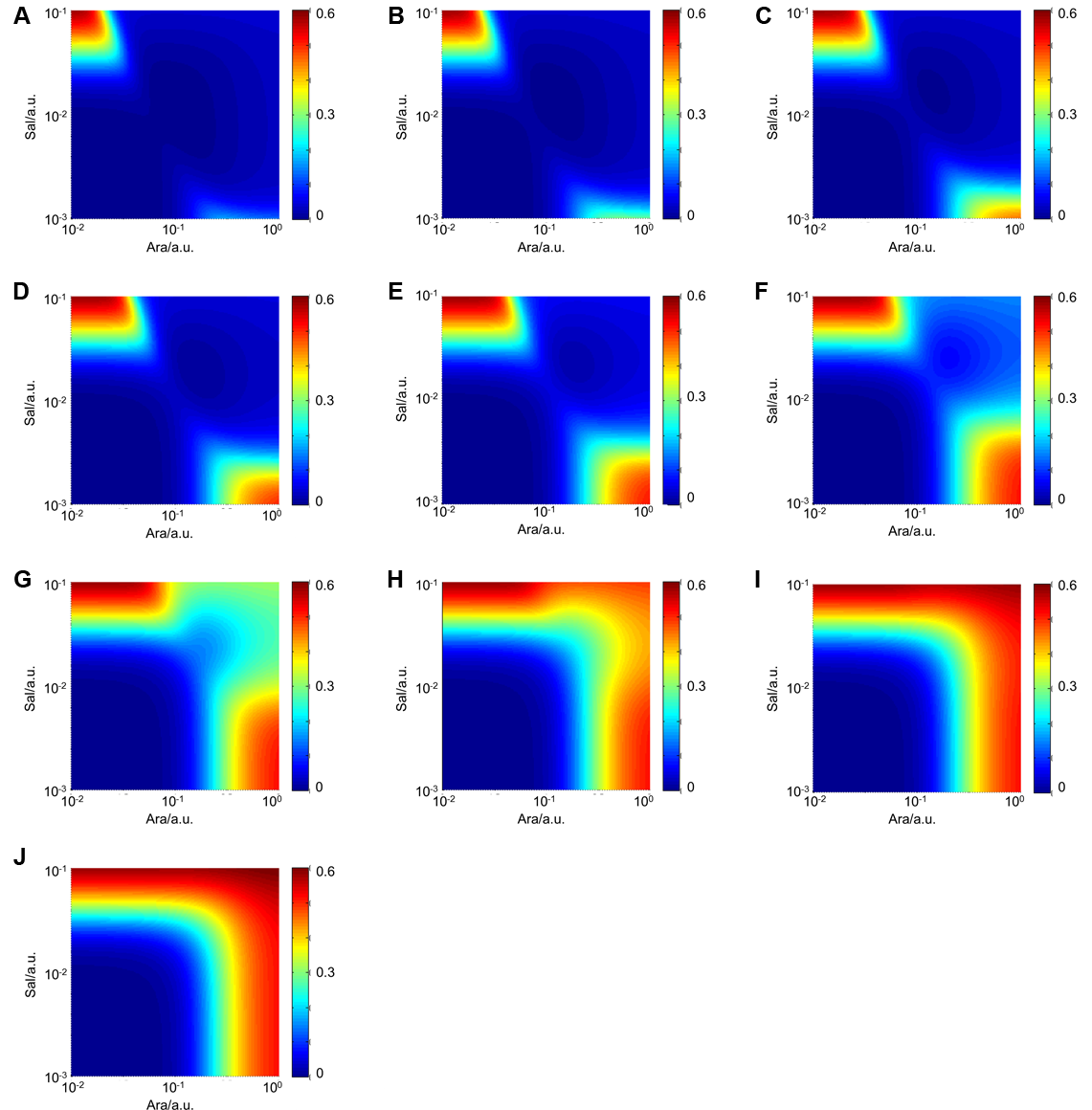

Supplement: Figure S8 — Model prediction for fine-tuning of circuiting interface. By adjusting luxI translation strength, different phase diagrams have been obtained. (A). Phase diagram for the original RBS, of which the sequence is AAAGAGGAGAAA. The other nine subfigures show phase diagrams with reduced translation strength in simulation. From (B) to (J), translation strengths are attenuated by 2, 5, 10, 20, 50, 100, 200, 500, and 1000 folds, respectively. And their corresponding signal-background ratios (the ratios of protein expression level between “ON” state and “OFF” state) are approximate 6, 9, 10, 8, 3, 2, 1, 0.9 and 0.8 in simulation. So we predict with modeling results that approximate 10-fold attenuated translation strength of RBS prefixing luxI would improve the circuiting. (TIF) [file pone.0057482.s008.tif]

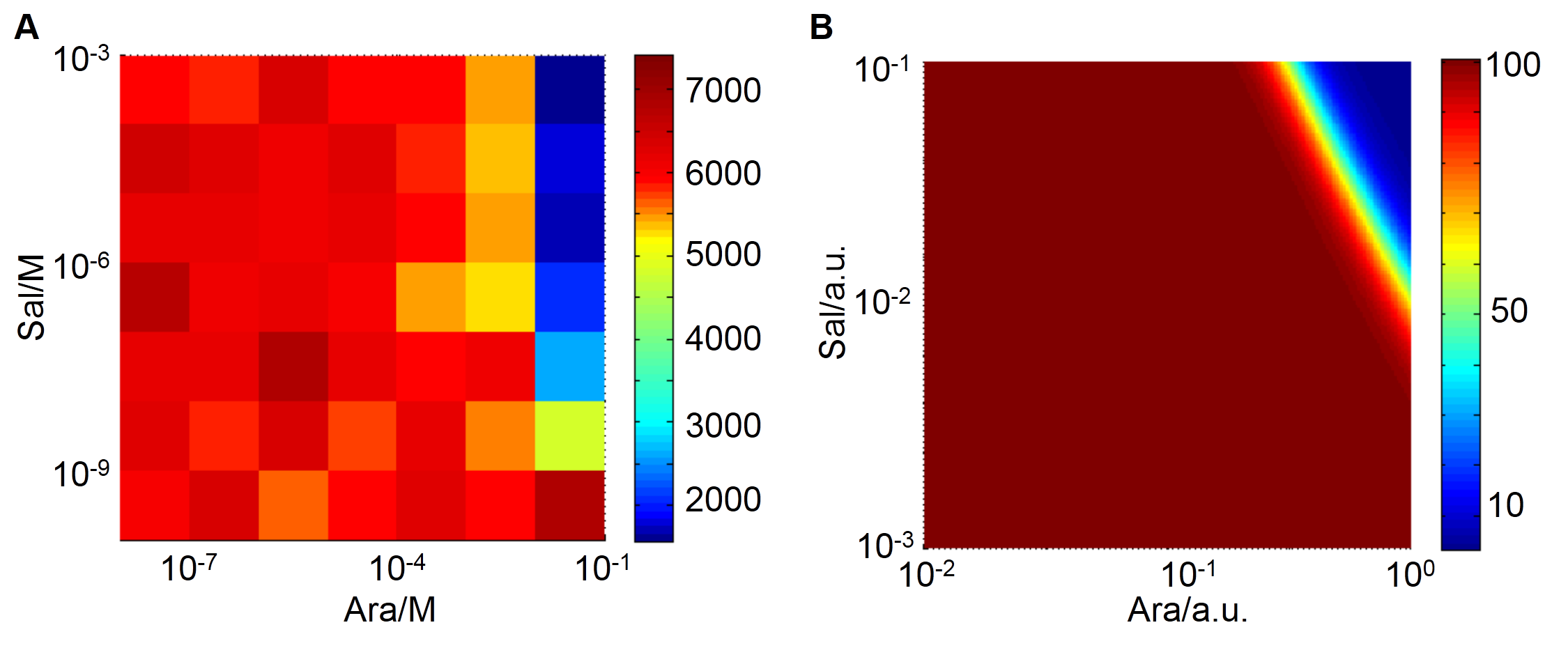

Supplement: Figure S9 — Transfer function of adapted USC with the RBS prefixing luxI changed to ATTAAAGTTGAGAAA. AHL levels are measured as the output. Experiment protocols are the same as described in the legend of Figure S6. The adapted USC still functions as an AND gate: only when both arabinose and salicylate exist, AHL would be expressed. (A). Experimental results. The data are for 10−1, 10−2, 10−3, 10−4, 10−5, 10−6, 10−7 and 10−8 M arabinose, and 10−3, 10−4, 10−5, 10−6, 10−7, 10−8, 10−9 and 10−10 M salicylate. (B). Corresponding simulation prediction. (TIF) [file pone.0057482.s009.tif]

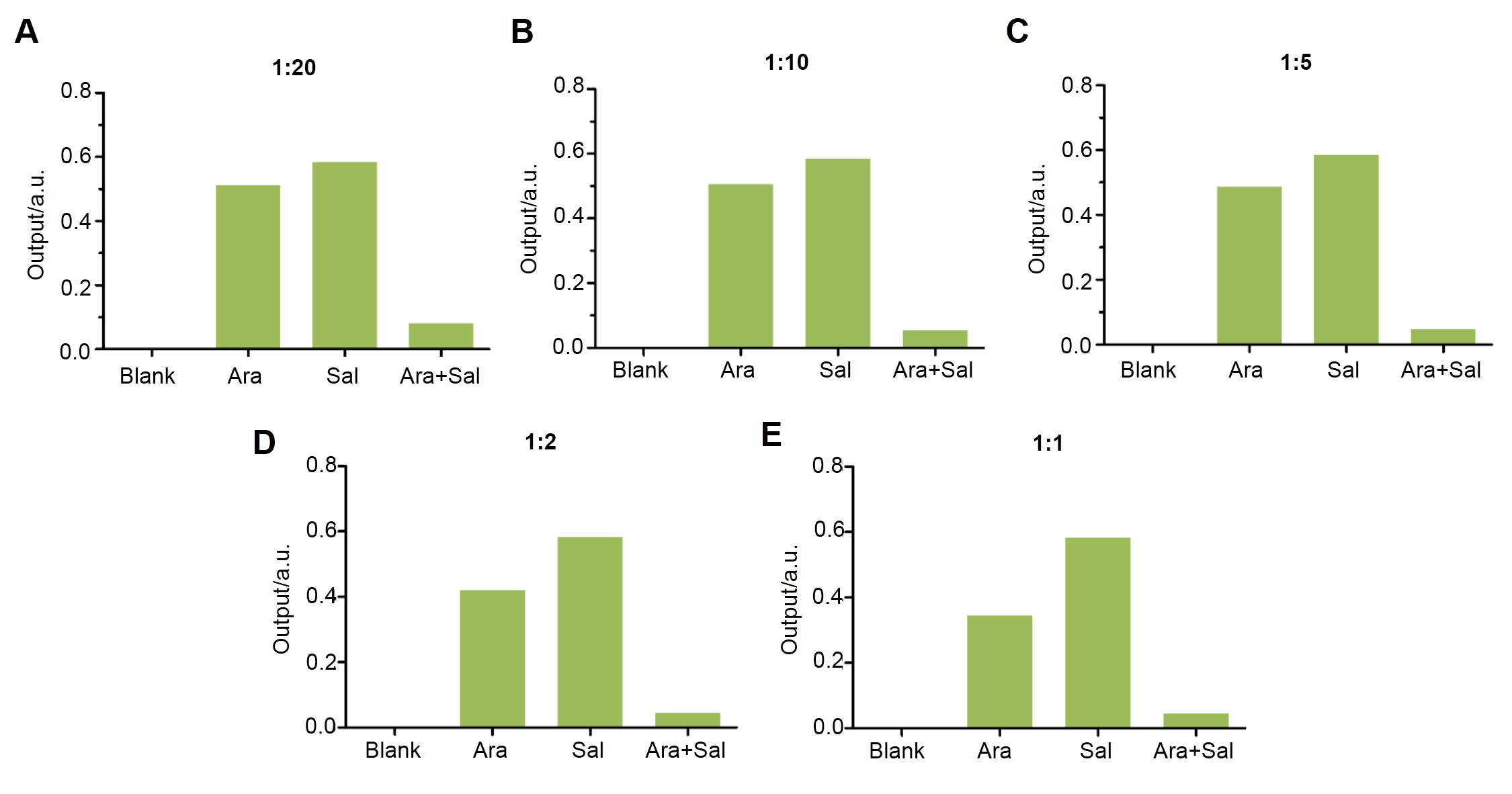

Supplement: Figure S10 — XOR gate operates robustly under population fluctuation in simulation. In simulation, we changed the population proportion of USC and DSC, and found that within a quite large range (from USC:DSC = 1∶20 to USC:DSC = 1∶1), the system could always exhibit a high signal-background ratio. (A). USC:DSC = 1∶20, (B). USC:DSC = 1∶10, (C). USC:DSC = 1∶5, (D). USC:DSC = 1∶2, and (E). USC:DSC = 1∶1. (TIF) [file pone.0057482.s010.tif]

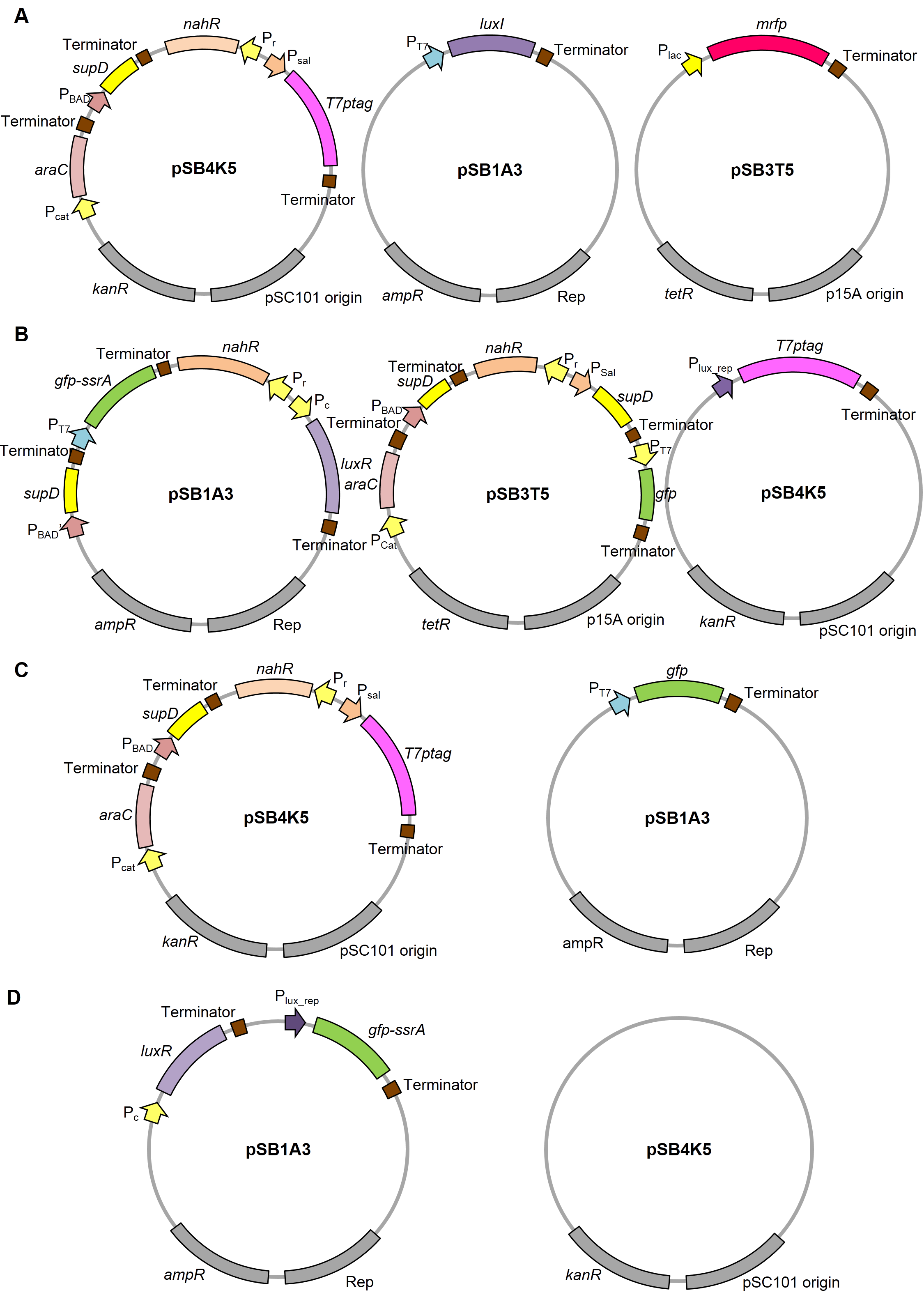

Supplement: Figure S11 — Plasmid constructions of USC and DSC. (A). Construction of USC for XOR function. The plasmid bearing RFP was used for flow cytometry assay. (B). Construction of DSC for XOR function. (C). Construction of USC used to measure its transfer functions. (D). Constructions used to measure AHL expression of USC. (TIF) [file pone.0057482.s011.tif]

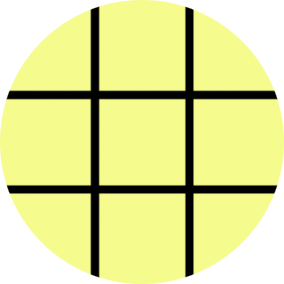

Supplement: Figure S12 — Plating and counting. When counting population proportions (in Methods), surface of agar plate was divided into 9 grids, of which 5 were selected for counting. We did not count the four regions at the corner with relative small area. (TIF) [file pone.0057482.s012.tif]
