# Supplementary material for: A Formalized Design Process for Bacterial Consortia That Perform Logic Computing
Source: PLoS One. 2013 Feb 28;8(2):e57482. doi: 10.1371/journal.pone.0057482 (PMC3585339; doi:10.1371/journal.pone.0057482)
Supplement: Table S1 — Truth tables for AND, OR and NOT gates. (PDF) [file pone.0057482.s013.pdf]

Inputs		Outputs		
**Input *A***	**Input *B***	***A* AND *B***	***A* OR *B***	**NOT *A***
0	0	0	0	1
0	1	0	1	1
1	0	0	1	0
1	1	1	1	0
