# Supplementary material for: A Formalized Design Process for Bacterial Consortia That Perform Logic Computing
Source: PLoS One. 2013 Feb 28;8(2):e57482. doi: 10.1371/journal.pone.0057482 (PMC3585339; doi:10.1371/journal.pone.0057482)
Supplement: Table S2 — Parameters and their corresponding values used in simulation. (PDF) [file pone.0057482.s014.pdf]

Parameter	Description	Value
**A**	Parameter in AND gate, see [1].	50±10
**B**	Parameter in AND gate, see [1].	3000±1000
**K_d**	Binding affinity of AHL to LuxR.	0.50±0.08
**K**	Binding affinity of LuxR to plux_rep.	0.03±0.01
**N**	Hill coefficient to describe the cooperativity effect of AHL	2.0±0.3
**I**	Strength of P_{lux_rep}	100±20
**C_0**	Translation strength of *luxI*	Varying from 0.01 to 100 with different RBS sequences
