# Supplementary material for: A Formalized Design Process for Bacterial Consortia That Perform Logic Computing
Source: PLoS One. 2013 Feb 28;8(2):e57482. doi: 10.1371/journal.pone.0057482 (PMC3585339; doi:10.1371/journal.pone.0057482)
Supplement: Table S3 — Primers designed for mutagenesis of Ribosome Binding Site sequence prefixing luxI . (PDF) [file pone.0057482.s015.pdf]

	Forward primers	Reverse primers
**Mutation 1**	GAGAAATACTAGATGACTAT AATGATAAAA	AACTTTAATCTCTAGTATCTCC CTATAGTGAG
**Mutation 2**	TCCCCGATGACTATAATGAT AAAAAAATCG	TGGAGCCTCTTTAATCTCTAGT ATCTCCC
**Mutation 3**	CCGATCATGACTATAATGAT AAAAAAATCG	AGGAGCCTCTTTAATCTCTAGT ATCTCCCTAT
