# Supplementary material for: A Formalized Design Process for Bacterial Consortia That Perform Logic Computing
Source: PLoS One. 2013 Feb 28;8(2):e57482. doi: 10.1371/journal.pone.0057482 (PMC3585339; doi:10.1371/journal.pone.0057482)
Supplement: Text S1 — Model for logic circuits, supplementary materials and methods. (PDF) [file pone.0057482.s016.pdf]

Supporting Information

A Formalized Design Process for Bacterial Consortia that Perform Logic Computing

Weiyue Ji[1#], Handuo Shi[1#], Haoqian Zhang[1,2#], Rui Sun[1], Jingyi Xi[1,2], Dingqiao Wen[1], Jingchen Feng[1], Yiwei Chen[1], Xiao Qin[1], Yanrong Ma[1] Wenhan Luo[1], Linna Deng[1], Hanchi Lin[1], Ruofan Yu[1] ,Qi Ouyang[2,3,*]

[1]Peking University Team for the International Genetically Engineered Machine Competition (iGEM), Peking University, Beijing, China, [2]Center for Quantitative Biology and Peking-Tsinghua Joint Center for Life Sciences, Beijing, China, [3]The State Key Laboratory for Artificial Microstructures and Mesoscopic Physics, School of Physics, Peking University, Beijing, China

* E-mail: qi@pku.edu.cn

# These authors contributed equally to this work and are listed in alphabetical order

Table of Contents

Model for logic circuits

Supplementary Materials

Supplementary Methods

Program code

Supplementary References

Model for logic circuits

Transfer function of genetic AND gate [1] is,

$$\frac{G}{G_{max}} = \frac{I_1 I_2{}^2}{a(b+I_2)^2 + I_1 I_2{}^2}, \qquad (1)$$

where G_{max} is the maximum fluorescence observed for the output, I_1 and I_2 should be the activity of input promoters upstream *T7ptag* and *supD* tRNA, respectively.

For OR gate，two promoters with the same coding sequence of downstream gene, the output of OR gate is additive, namely,

$$G = k(I_1 + I_2), \qquad (2)$$

where G is output of OR gate.

As for NOT gate (quorum sensing repressors), the binding of a ligand to its transcription factor at equilibrium is,

$$C = C_0 \frac{L^n}{K_d{}^n + L^n}, \qquad (3)$$

where C is the concentration of bound transcription factor, C_0 is the total concentration of transcription factor, L for the concentration of ligand, K_d for dissociation constant, and n for cooperative index. By mass conservation, the concentration of free transcription factor C_F is,

$$C_F = C_0 - C. \qquad (4)$$

The probability for each promoter in open is described by the following equation:

$$P = \frac{1}{1 + C_F/k}. \qquad (5)$$

Now we can combine the above equations together and see how our XOR gate works.

For USC, as in Eq. (1), $G = G_{max} \frac{I_1 I_2{}^2}{a(b+I_2)^2 + I_1 I_2{}^2}$, and assume that the amount of AHL is proportional to LuxI. Therefore the concentration of AHL should be

$$A = A_{max} \frac{I_1 I_2{}^2}{a(b+I_2)^2 + I_1 I_2{}^2}. \qquad (6)$$

AHL would bind to its transcriptional factor and the concentration of bound transcription factor at equilibrium is (Eq. 3),

$$C = C_0 \frac{A^n}{K_d{}^n + A^n}. \qquad (7)$$

Further, activity of P_{lux_rep} could be derived through Eq. 5,

$$P_{lux\_rep} = \frac{I}{1 + C_F/k}. \qquad (8)$$

Now, for the second AND gate in DSC, we can define the two inputs as,
$I_1' = P_{lux\_rep}$,
$I_2' = I_1 + I_2$.

Finally, we obtain, $OUTPUT = OUTPUT_{max} \frac{I_1' I_2'^2}{a(b+I_2')^2 + I_1' I_2'^2}$.

All parameters utilized in model are summarized in Table S2, by fitting experimental data with our model. Errors are calculated as s. d..

Supplementary Materials

No.	Part Design	Part Number
**1**	AND GATE (AraC+P_{BAD}+SupD+NahR+P_{Sal}+RBS(B0033)+T7ptag)	BBa_K228258
**2**	T7 Promoter	BBa_I719005
**3**	AHL Synthase, RBS+luxI	BBa_C0261
**4**	RFP Coding Device(P_{lac}+RBS+RFP+terminator)	BBa_J04450
**5**	P_{BAD} strong	BBa_K206000
**6**	SupD-tRNA	BBa_K228001
**7**	Double terminator	BBa_B0015
**8**	RBS+GFPssrA	BBa_K581007
**9**	RBS + LuxR	BBa_J37033
**10**	AraC regulatory protein (P_{Cat}+AraC)	BBa_K228008
**11**	P_{BAD} promoter	BBa_I13453
**12**	NahR(reverse) - salicylate promoter	BBa_K228004
**13**	T7GFPmut3b (P_{T7}+RBS+GFP+terminator)	BBa_I719015
**14**	Promoter (HSL-mediated luxR repressor)	BBa_R0061
**15**	T7ptag(T7polymerase with amber mutation)	BBa_K228000
**16**	GFP generator	BBa_E0840

All parts were obtained from Registry of Standard Biological Parts (http://partsregistry.org/Main_Page), with their number indicated in above table.

Supplementary Methods

Mutagenesis of Ribosome Binding Site (RBS) sequence prefixing *luxI*. Site-directed mutagenesis was conducted using MutanBEST Kit (Takara BIO INC) following the manufacturer's protocol. A pair of mutation-creating primers was designed with their 5' ends adjacent and 3' ends in opposite directions. The entire pSB1A3 plasmid bearing RBS-*luxI* was PCR- amplified using the designed primers,

producing blunted products. Resulted product was 5'-phosphorylated, self-ligated, transformed to E. coli strain DH5α and plated. Primers are explicitly shown in Table S2.

Program code

Program code for Logic Gate assembly and design are available in http://sharesend.com/vz9de.

We also provide compiled version of our software, which can be accessed from http://sharesend.com/q7iyi (Windows) and http://sharesend.com/0pqvu (Linux).

Supplementary References

[1] J. C. Anderson, C. A. Voigt and A. P. Arkin, Environmental signal integration by a modular AND gate, *Mol. Syst. Biol.*, 2007, **3**, 133.
